# Observations on Phthisis in Martinique

**Published:** 1844-10-01

**Authors:** 


### Observations on Phthisis in Martinique. By *E. Rufz*, M.D.

In reviewing the tenth volume of the "Memoires de l'Académie Royale de Médecine," some time since, we inadvertently passed over this paper, which contains some points of sufficient interest to justify a brief analysis.

The Academy having inquired the opinion of the practitioners in the French colonies as to the prevalence of phthisis in those localities, Dr. Rufz forwarded the above paper. It contains his experience at St. Peter's, Martinique, for four years, during which time he attended in private practice 1954 patients, of whom 123 or about 13 per cent, were cases of phthisis. Other practitioners, living in various parts of the island, whom he interrogated, represented the disease as prevalent as he found it, constituting the most frequent of all the chronic maladies. It is remarkable that other chest affections are very rare, so that during five years the author saw but three cases of pneumonia, while chronic bronchitis is just as seldom seen even in aged persons. Other affections, again, which are usually considered as belonging to the same family as phthisis, as white-swelling, Pott's disease, glandular engorgements, &c. are seldom met with.

*Hæmoptysis.*—In seven out of the nine cases in which an autopsy was made, the patient succumbed under excessive hæmoptysis, a termination which, according to Louis and Chomel, is rare in Europe. In no case could any erosion of a vessel be detected, and even when the bronchi, trachea, and stomach were filled with blood, the excavations in the lungs contained neither fluid or coagulated blood. In 55 fatal cases, hæmoptysis, in a greater or less degree, was noted 39 times, reproduced in the majority of cases at various stages of the malady. It did not seem to hasten the progress of the disease. The entire number of cases of hæmoptysis the author has met with in four years is 63, and of these 51 were evidently cases of phthisis.

*Abdominal Lesions.*—Of nine cases examined after death, in only two were ulcerations of the intestinal canal discovered, and these were very slight. This would be expected from the fact, that *diarrhœa,* even in the last stage, is a very rare complication in Martinique. The fact is the more singular, as the gastro-intestinal functions are very susceptible of derangement in that island, constituting the great mass of cases of disease, while the treatment of phthisis by purgative medicines is a very popular practice.

*Age.*—The author gives this table—

| | |
|---|---|
| From 10 to 13 years | 2 |
| —— 15 ,, 20 ,, | 14 |
| —— 20 ,, 30 ,, | 39 |
| —— 30 ,, 40 ,, | 25 |
| —— 40 ,, 60 ,, | 4 |
| After 60 | 9 |

The freedom of *young children* from phthisis is remarkable in Martinique, for Dr. Rufz, in all the autopsies he has made for various diseases in children, has never met with tubercles but twice; although most of these cases had sank in consequence of prolonged diarrhœa, the most fatal complaint of children in that climate. He explains it thus:—

"Perhaps we may find the cause of this in the mild temperature of the climate, so favorable to the early years of life, and in the various habits consequent upon such a temperature. Children during the first months are only clothed in a linen shirt, and sometimes are allowed to go naked. They are not confined in cradles, or even within doors, but are placed upon a straw mat in the open air, and allowed to kick about as they please. About six or eight months after birth they crawl about on their hands and feet, and at ten months or a year old walk firmly. In the town of St. Peters, where running water is found in every house, they often bathe themselves two or three times a day. Nevertheless, such is the influence of warmth in infancy, than when these children are debilitated by diarrhœa or other cause, it is found best, notwithstanding the heat of the country, to clothe them in a flannel shirt.

"After the age of six or seven years, these children, so beautiful at first that an European, accustomed to the children of the North, always believes them six or eight months older than they are, undergo a change, become pale or yellow; and it is rare to find the same strong and healthy adolescence we meet with in the schools in France. It is at this period a voyage to Europe is so useful. The same difference exists even in the negro, although it is less sensible; the negro infants being a much less fine race than those of the French peasants."

The author is disposed to believe that the climate of Martinique exerts a retarding effect occasionally upon the progress of consumption. He has met with eight cases which, notwithstanding marked signs of the disease at an early period of life, have reached old age.

*Sex.*—Forty-five men and fifty-six women were the proportions observed by Dr. Rufz ; and in this case, *stays* exert no influence in giving the usual preponderance to females, since they are unknown in Martinique, where the garments are light and ample.

*Constitution.*—Among the women, 44 are noted as of feeble, and six of strong constitution; and among the men, 30 are of feeble, and 12 of strong constitution. The author observes, it is a great error to suppose that weak constitutions are those best adapted for a residence in warm climates. Persons of robust frame and temperate habits best resist the effects of the torrid zone.

*Race.*—These colonies have been peopled by Europeans and Africans. Children born of Europeans intermarrying, are white, and are termed white creoles. The children of Africans are termed black creoles. The union of Europeans with Africans has produced a population of various shades known as *mixed-bloods.* Of these last, the *mulatto* results from the union of the white and black, and the *capre* from that of the black and mulatto. The following is the proportion in which phthisis has manifested itself in each.

| | |
|---|---|
| European Whites | 3 |
| White Creoles | 49 |
| Mulattos | 22 |
| Capres | 7 |
| African Negros | 2 |
| Negro Creoles | 19 |

Thus, the disease is rare among Europeans. The same fact is shewn by the experience of the military hospital of St. Peters, containing only European soldiers, from the age of 18 to 30; and from which it seems that, out of 400 patients admitted for various diseases, two or three only were affected with phthisis. The author contrasts this with what he had observed in the military hospitals of the South of France, where such cases abound. The proportion of the white creoles may seem unduly great in this table, as the practice of Dr. Rufz lies principally among that class. Phthisis proves especially fatal to the mulatto-women, who furnish the victims for public prostitution. The negro-creoles, considering that they are three times as numerous as the whites and mulattos united, offer a small proportion of cases of phthisis. As the slave-trade has been abolished since 1830, the number of African negros, of the age especially liable to phthisis, is but few.

Seeing that the disease is so rare among the two or three hundred soldiers who are sent annually from Europe to replenish the garrison, exposed as they are to various debilitating causes, as nostalgia, intermittent fever, dysentery, &c., and how few resident Europeans are attacked by it, one is tempted to believe the climate of these Colonies is favorable to the disease. On the other hand, it is to be observed, that many Creoles who have remained during a long period in France for the purposes of their education, become the victims of phthisis very shortly after their arrival, which leads to the belief that the climate cannot be favorable to those already predisposed.

*Hérédité.*—Pursuing his enquiries into thirty families in which phthisis had prevailed, Dr. Rufz found that, in three instances, the fathers had died phthisical, in five the mothers, in two the uncle or aunt—eleven times a greater or less number of brothers or sisters, and three times the cousins. It would seem that phthisis is less a hereditary disease, transmissible from father to son, than a congenital defect peculiar to children issuing from the same blood.

*Temperature.*—The suppression of perspiration by chills is the most powerful cause of disease in Martinique. The vicissitudes of temperature are by no means so considerable as in Europe. The thermometer is never raised at St. Peter's above 24° Reaumur, and, while thus high, seldom falls lower than 20°. But, although the greatest variation is not more than three or four degrees, bodies highly heated easily lose a large quantity of caloric, and slight breezes affect as much as thermometrical variations. It is not rare, upon the hottest days, to feel oneself as if frozen, while bathed in perspiration ; and a short sleep is often followed by this sense of chilliness. So that the use of the flannel-waistcoat, for the purpose of absorbing the superfluous sweat, and preventing the ill effects of clothing soaked with it being in contact with the skin, is more common in this colony than in any country in the world.

*Treatment.*—Dr. Rufz states, that a greater success attends this in Martinique than in Europe. He adopts the same plans of treatment that there prevail. Among hygienic procedures he especially recommends *horse-exercise;* and although he cannot go so far as Sydenham, in terming this the bark of phthisis, he has always derived good effects from its employment. One advantage of the climate of Martinique is, that the patient may take exercise out of doors during any part of the year; and the author has seen patients avail themselves of this solace until the last day of their existence.

It is interesting, in connexion with the recommendation of their employment by the high authority of Sir James Clark in our own country, to find the author a warm advocate for the more frequent use of *emetics* in the treatment of phthisis. In many cases in which he has had recourse to them, they have produced very beneficial effects in quieting the cough, calming the respiration, and improving the appetite. He has given them in all stages, and only on three occasions has found it necessary to suspend their use by reason of the fatigue they caused. In two cases they arrested hæmoptysis which had resisted other measures. He employs ipecacuanha or antimony indifferently.

